# Contingency Management for Veteran Smokers Undergoing Elective Major Surgery: Protocol and Rationale for a Pilot Randomized Controlled Trial

**DOI:** 10.2196/97779

**Published:** 2026-09-10

**Authors:** Ellen Herbst, Christy Yim-Fan Wong, Elliott Chaney, Theodore Fetterling, Isaac A Mirzadegan, Phiroz E Tarapore, Benjamin Emmert-Aronson, Gabriela K Khazanov, Katherine J Hoggatt, Madeline Martinez Rivas, Sadaf Dabiri, Jayabhargav Annam, Julia M Harris, Lauren Paver Thompson, Brian Borsari

**Affiliations:** 1 Department of Psychiatry and Behavioral Sciences, University of California, San Francisco, 675 18th St., San Francisco, CA, United States, 1 415-221-4810 ext 26078; 2 Center for Data to Discovery and Delivery Innovation (3DI), San Francisco VA Health Care System, San Francisco, CA, United States; 3 Mental Health Service, San Francisco VA Health Care System, San Francisco, United States; 4 Research Service, San Francisco VA Health Care System, San Francisco, United States; 5 Department of Neurological Surgery, University of California, San Francisco, San Francisco, CA, United States; 6 Surgical Service, San Francisco VA Health Care System, San Francisco, United States; 7 Ferkauf Graduate School of Psychology, Yeshiva University, New York, NY, United States; 8 Department of Medicine, University of California, San Francisco, San Francisco, CA, United States; 9 Department of Psychiatry, Kaiser Permanente, Oakland, CA, United States; 10 VA Quality Scholars Advanced Fellowship Program, San Francisco VA Health Care System, San Francisco, CA, United States; 11 San Francisco VA Medical Center, Trailer 37, Room 04, 4150 Clement St.San Francisco, CA, 94121, United States, 1 415-221-4810 x26078, 1 415-750-6987

**Keywords:** contingency management, mobile health, nicotine, motivational interviewing, ecological momentary intervention, smoking cessation, perioperative smoking cessation, tobacco use disorder

## Abstract

**Background:**

Smoking is the leading risk factor for postoperative complications and is associated with longer hospital stays, reoperations, and 30-day mortality. Smoking rates among patients undergoing major elective surgery are high (22.3%‐43.0%). It is urgent to identify efficacious, strategically timed smoking cessation interventions for patients undergoing surgery.

**Objective:**

The goal of this pilot randomized controlled trial is to design and test the feasibility of the first mobile contingency management (mCM) smoking-cessation intervention for military veterans undergoing major elective surgery and to evaluate the effectiveness of mCM compared to treatment as usual (TAU) as exploratory analysis.

**Methods:**

We will adapt and adjust a contingency management protocol for delivery over 5 weeks using mobile carbon monoxide monitoring in the perioperative period. We will then test its feasibility and effectiveness in veteran smokers undergoing major elective surgery (n=36). Participants will be randomized to receive mCM or TAU. Both groups will receive pharmacotherapy. The feasibility of mCM will be assessed by (1) recruitment rate, (2) level of engagement, and (3) retention. We will also compare mCM with TAU on smoking cessation outcomes (quit attempts, cigarettes per day, and nicotine dependence severity) over 5 weeks to examine effectiveness.

**Results:**

This pilot trial has been funded since July 2024, and a 1-year no-cost extension has been granted through June 2027. The recruitment and data collection started in December 2025. The first participant consented on December 12, 2026. As of May 2026, 18 participants have been enrolled and randomized in this pilot trial. Enrollment of the last participant is expected to be in March 2027 to reach a target of 36 enrolled participants, and the data collection is expected to be completed by April 2027. There will be no interim data analysis, but preliminary analysis is expected to be completed by August 2027, and all data analyses will be completed by the end of 2027.

**Conclusions:**

Novel interventions to support smoking cessation may reduce morbidity, mortality, and health care costs for patients undergoing major surgery. mCM has a high potential to implement widely to improve surgical outcomes.

## Introduction

### Background

Major surgery is common among veterans, and smoking is the most significant modifiable risk factor for perioperative morbidity and mortality [1]. The Veterans Affairs (VA) Health Care System performs over 600,000 major elective surgeries annually, including general, thoracic, vascular, urologic, spinal, and other orthopedic procedures [2]. Unfortunately, smoking prevalence among patients undergoing major elective surgery is high, ranging between 22.3% and 43.0%, and is highest among uninsured patients (36.3%) and Medicaid recipients (43.0%) [3]. Reducing complications and death after major elective surgery is a top priority for both veterans [1] and the general population [3]. Smoking-related postoperative risks include pneumonia, prolonged intubation, myocardial infarction, and stroke [4-6]; infections, sepsis, and poor wound healing [4-9]; and repeat procedures [4-9]. Active smoking status at the time of surgery is the single greatest risk factor for complications, reoperation, and death [4-9]. The relative risk (RR) of death in 30 days following major surgery is 1.5 times higher in smokers than in nonsmokers [8,9]. Accordingly, the American College of Surgeons recommends that smoking cessation counseling be offered during all nonemergent consultations [9].

The preoperative period offers a critical opportunity to promote behavior change [3,10,11]. A systematic review of clinical trials found that intensive perioperative smoking cessation programs, with sessions before and after surgery, increased the likelihood of postoperative abstinence in both the short term (odds ratio 2.41, 95% CI 1.95-2.98) and the long term (odds ratio 1.64, 95% CI 1.23-2.20) [10]. Another systematic review and meta-analysis examining rehabilitation interventions for behavioral change concluded that participation in 4-week behavioral interventions before surgery was associated with higher odds of cessation (RR 2.9, 95% CI 1.7-4.8; *I*^2^=84%) and sustained abstinence at 12 months post surgery (RR 1.74, 95% CI 1.20-2.55; *I*^2^=43%) [11]. Taken together, intensive smoking cessation interventions before and after surgery can improve short-term and long-term quit rates.

Contingency management (CM) is a behavioral treatment that uses positive reinforcement to incentivize behavior change through financial rewards (ie, vouchers and cash equivalent) when the target behavior (eg, smoking cessation) is achieved and biologically verified. CM interventions are associated with rapid reductions in substance use during the active delivery of incentives [12,13] and are effective for a range of substance use disorders (SUDs), including tobacco use disorder (TUD) [14-18], in both men and women [19]. A Cochrane review examining CM for smoking abstinence compared with controls reported a pooled risk ratio of 1.49 at the longest follow‐up (6 mo or more) [20]. CM is commonly combined with other evidence-based treatments for TUD such as pharmacotherapy and behavioral counseling [21] and can be strategically timed to promote abstinence at a crucial period when the clinical benefit of cessation is substantial. The VA is a leader in implementing CM for a range of SUD [22,23], delivering CM to over 7700 veterans across 134 sites since 2011. While CM for stimulant use disorder is well established in the VA, CM for smoking cessation is in the earlier stages of implementation.

A fixed-incentive CM structure is flexible and suited to remote delivery, while the optimal frequency, timing, and duration of financial incentives to promote durable, long-term abstinence are areas of active inquiry [24]. CM incorporates clinician feedback at the time of bioverification and incentive; feedback is brief, focused on the target behavior, and includes praise for abstinence, expressions of empathy and confidence, assessment of the target behavior and craving, and encouragement of follow-up [25].

Attendance and retention are key predictors of CM outcomes; thus, CM protocols for smoking cessation must include replicable, consistent bioverification [20,25-28]. Advances in digital health have facilitated remote CM bioverification and monitoring of smoking abstinence using a personal, mobile carbon monoxide (CO) device at home. A recent meta-analysis concluded that CM delivered via telehealth is an efficacious intervention for SUD, including TUD [14] (pooled effect sizes for the percentage of negative samples [*d*=0.94], longest duration of abstinence [*d*=1.08], and quit rate [*d*=0.46]). In clinical research, mobile contingency management (mCM) for smoking cessation with CO bioverification has shown benefits in different veteran populations [14,20], including those in rural areas [29], those who are unhoused [27], and those with SUD [30] and posttraumatic stress disorder [31]. CM is safe; problem gambling is the only relative contraindication to CM, due to the potential to reactivate gambling behaviors [32]. CM has numerous advantages for veterans undergoing major surgery, given its association with rapid reduction in tobacco use, its ability to be strategically timed for the highest impact, its excellent safety profile with few contraindications, and its scalability within the VA system. CM for TUD delivered perioperatively may prolong lifespan, reduce complications, cut health care expenditures, and enhance quality of life.

To our knowledge, only 1 randomized controlled trial (RCT) [33] has tested CM for smoking in the perioperative setting; CM predicted more rapid abstinence, a lower percentage of positive tests, and fewer lapses following abstinence [33]. However, gaps exist in terms of feasibility and methodological considerations, given that this CM protocol in the aforementioned RCT did not employ bioverification 5 to 7 days per week to verify abstinence and was conducted in a surgical cohort with cancer. No trials that we are aware of have examined CM for a broader population of patients undergoing major elective surgery, delivered remotely, or examined a more rigorous CM schedule to bioverify abstinence.

The present proof-of-principle pilot study addresses a critical gap in perioperative care by providing a scalable, digital CM solution that meets the specific needs of veterans and other high-priority groups. Although CM for TUD has strong empirical evidence, it has never been trialed before and after major surgery nor tested in a VA context. Moreover, no smoking-cessation RCT of CM with near-daily mobile bioverification—now considered a first-line approach [26,27]—has been conducted with patients undergoing major elective surgery. Research indicated that behavioral interventions for smoking cessation before surgery [11] and continuation after surgery [10] are associated with favorable outcomes [24]. This investigation aims to establish mCM as an effective approach for perioperative smoking cessation, with the potential to inform broader CM applications.

### Study Objectives and Aims

In this pilot RCT, veteran smokers undergoing major elective surgery (n=36) will be randomized to receive the 5-week mCM protocol or 5 weeks of treatment as usual (TAU; offering VA Tobacco Cessation Clinic and VA Telephone Quitline) remotely. The mCM smoking-cessation protocol will comprise a 5-week, fixed-incentive schedule, including once-daily CM incentives contingent on bioverified abstinence, weekly behavioral coaching, and remote CO monitoring. Both groups will be offered smoking-cessation medication [21]. Our first aim (aim 1) is to develop an mCM smoking-cessation protocol to be integrated into VA surgical settings. This protocol will modify an extant CM model for smoking cessation within the VA [34] to be adapted for delivery via telehealth. Additional aims include assessing the feasibility of CM for TUD in surgical settings (aim 2) and comparing CM with TAU (exploratory aim). We predict that CM will be feasible in the surgical setting, as assessed by measures of enrollment, retention, and engagement in the CM sessions. As exploratory outcomes, we will explore the effect of CM versus TAU on smoking cessation and abstinence outcomes.

## Methods

### Participants and Eligibility

Inclusion criteria are as follows: (1) veterans enrolled in the San Francisco VA Health Care System (SFVAHCS), (2) those recommended or scheduled for major elective surgery within the next 6 months (eg, general, spinal, thoracic, vascular, urological, gynecological, orthopedic, or other surgeries), (3) those currently (ie, past 30 d) smoking a minimum average of 1 cigarette per day, (4) those having daily access to a personal smartphone device and Wi-Fi, and (5) those open to receiving smoking-cessation interventions. Veterans followed by primary care who are referred to external institutions for major elective surgery will also be eligible.

Exclusion criteria will be evaluated through medical record review and clinical assessment: (1) psychotic, bipolar, or neurocognitive disorders or other psychiatric or medical conditions judged to be unstable in the past 30 days; (2) untreated, current, active problem gambling, as assessed by a medical record diagnosis or a Problem Gambling Severity Index (PGSI) score ≥8; (3) enrollment in end-of-life or palliative care; (4) surgery with a clinical indication for cancer; (5) unable to meet time commitment; (6) currently pregnant or planning to become pregnant during the study [21]; (7) a suicide attempt, or suicidal ideation with intent, within 30 days prior to enrollment; and (8) concurrent participation in another tobacco cessation trial.

### Recruitment and Overall Study Schedule

Participants will be recruited through (1) data queries of VA electronic health record data available through the VA Corporate Data Warehouse and (2) direct recruitment. Data queries will identify patients with an upcoming major elective surgical procedure from a predefined list. We will then identify individuals with documentation of current smoking, using an established approach via the Corporate Data Warehouse national VA database with codes, or “health factors.”

Partners in surgical subspecialties will assist with recruitment through referrals. Direct recruitment will allow the team to recruit participants with a stated interest in tobacco cessation and facilitate targeted recruitment in clinics that serve women and members of underrepresented minority groups. If recruitment rates decrease to below 2 participants per month, we will expand the eligibility criteria from major surgeries to include less invasive eye surgeries and podiatric surgeries.

The protocol eligibility criteria were developed to include participants up to 6 months prior to surgery to improve generalizability after extensive discussions with colleagues in surgery. Surgical colleagues described that some elective major surgeries (eg, some orthopedic, vascular, and neurosurgeries) may not be scheduled for months until successful smoking cessation has occurred, given the risks of cigarette smoking and impaired wound healing with some procedures. Therefore, to be as inclusive as possible to a range of participants awaiting scheduling for elective major surgery, the eligibility criteria include participants who smoke up to 6 months prior to surgery. The preoperative period varies by participant and is being tracked in this pilot study.

Once a veteran participant is recruited, we will follow the study timeline as delineated in Table 1. This schedule of enrollment, interventions, and assessments adheres to the latest SPIRIT (Standard Protocol Items: Recommendations for Interventional Trials) guidelines for reporting (Checklist 1) [35].

**Table 1.** Schedule of enrollment, interventions, and assessments.

	Trial period							
	Enrollment		Postrandomization					Closeout
	Screening and baseline		Intervention phase					Follow-up
Time point (wk)	Week −4	Week 0^a^	Week 1	Week 2	Week 3	Week 4	Week 5	Week 6^a^
Time window (d)^b^	30	7±14	7	7	7	7	7	7+14
Enrollment								
Eligibility screen	✓							
Informed consent	✓							
Medication evaluation		✓				✓		
Randomization		✓						
Postrandomization—intervention or comparator								
Contingency management (CM)			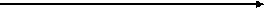					
Treatment as usual (TAU)			✓	✓	✓	✓	✓	
Assessments								
Medical record review	✓	✓						✓
Concomitant medical and mental health treatment	✓	✓						✓
Kessler Psychological Distress Scale	✓							
Columbia-Suicide Severity Rating Scale	✓							
Timeline Followback (TLFB)^c^	✓							✓
Problem Gambling Severity Index	✓							
Urine pregnancy test^d^	✓							
Demographics Questionnaire		✓						
Nicotine and Tobacco Use Survey		✓						✓
Fagerström Nicotine Dependence Questionnaire		✓						✓
Contemplation Ladder—Tobacco Smoking		✓						✓
Questionnaire of Smoking Urges (QSU)^e^		✓						✓
12-Item Short Form Health Index		✓						✓
Exhaled carbon monoxide (CO)								✓
Salivary cotinine^f^								✓

a Week 0 and week 6 can be completed within a 14-day window after the scheduled week. Participants can start week 0 (baseline) earlier, that is, −14 days, if the process is efficient in completing the screening and baseline tasks; or the +14 day window can be used to complete all baseline assessments. At week 6, the typical week 6 range is 7 days, but we allow up to 14 days after week 6 to complete all follow-up measures, if needed.

b Given that the study population includes patients with a high degree of medical complexity and comorbidities, dates are approximate, and assessments can occur on alternate dates if the patient is managing medical appointments that conflict with assessment time points (including but not limited to, chemotherapy, radiation, hospitalization, etc). Alternate scheduling to account for medical comorbidities will occur at the discretion of the principal investigator and co-investigators.

c Cigarette smoking might be reassessed in week 0 to ensure data collected covered past 30 days prior to randomization/intervention phase. Time frames for TLFB in general are past 30-day for screening, past 7-day at weekly session, and days between last TLFB assessment and follow-up assessment for CM condition; and past 30-day for screening and days between the last TLFB assessment and follow-up assessment for TAU condition. TLFB will cover all the days since 30-day prior to screening to the last day of week 6.

d Only individuals aged 18 to 55 years with childbearing potential will be required to take a urine pregnancy test.

e One item from the QSU will be used for assessing craving weekly during CM intervention. This has been specified in Table 2.

f Salivary cotinine will be used as needed to resolve the inconsistency between the CO results. Individuals with CO levels >6 ppm, who report smoking cannabis via TLFB and are not taking any formulation of nicotine, salivary cotinine <10 ng/ml will be used.

**Table 2.** Mobile contingency management (mCM) intervention protocol.

mCM components	Week				
	1	2	3	4	5
iCO Smokerlyzer^a^/iCO app training (daily, weekend, and holidays optional)	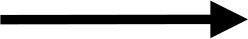				
iCO Smokerlyzer/CO video uploads (daily, weekends, and holidays optional)		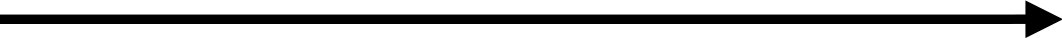			
CM^b^ incentive (daily)	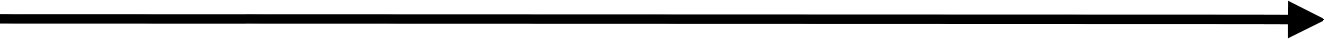				
Behavioral counseling and clinician feedback session (once weekly)	✓	✓	✓	✓	✓
Assessments^c^					
Timeline Followback (TLFB)	✓	✓	✓	✓	✓
Questionnaire of Smoking Urges (QSU)^d^	✓	✓	✓	✓	✓

a The Smokerlyzer (coVita) is a breath carbon monoxide (CO) device that aids smoking cessation by measuring the amount of CO in the breath of a smoker.

b CM: contingency management.

c Assessments (TLFB and QSU) will be administered weekly to assess past 7-day smoking and the level of craving.

d One item from the QSU will be used for assessing craving weekly.

### Randomization and Blinding

Eligible participants will be randomly assigned to either CM or TAU in a 1:1 ratio, stratified by (1) biological sex and (2) cigarettes per day (≥10 or <10 cigarettes per day) using random permuted blocks via REDCap. We will examine subgroups in exploratory analyses. Due to staffing constraints, no member of the study team will be blinded.

### Measures and Data Collection

#### Demographic, Exclusionary, and Baseline-Only Measures

Demographic characteristics will be collected at baseline. Participants will complete the PGSI [36], a validated 9-item self-report measure of problem gambling behaviors, where a score of ≥8 indicates problem gambling, and Kessler Psychological Distress Scale [37], a validated 10-item survey that measures emotional states, in which a score of 30 and over is likely an indication of severe mental problem. The principal investigator (PI) will assess participants’ past-30-day suicidality via Columbia-Suicide Severity Rating Scale per VA suicide screening procedures [38] and conduct a thorough chart review to confirm mental stability for participating in the study.

#### Outcome Measures

##### Smoking Measures

Smoking cessation outcomes will be administered at baseline and posttreatment. A Nicotine and Tobacco Use Survey (NTUS) developed internally will assess age at first use, duration of use, use of all forms of nicotine and tobacco, prior quit attempts (ie, intentionally not smoking for ≥24 h), duration of cessation (if any), and presence of other tobacco users in the home. Posttreatment assessment will include frequency and duration of quit attempts in the past 30 days. The Fagerström Test for Nicotine Dependence (FTND) [39] is a validated instrument of the severity of physiological addiction to nicotine. The Contemplation Ladder [40] is a validated single-item measure of motivation to quit smoking. The Questionnaire of Smoking Urges [41,42] is a validated 10-item measure that assesses cigarette craving and withdrawal. It will also be administered weekly to assess craving via 1 item, *“*I have a desire for a cigarette right now,” via a scale from 0 (strongly disagree) to 100 (strongly agree) [42]. The Timeline Followback (TLFB) [43,44] is a validated, staff-administered instrument that uses anchor dates to prompt recall of substance use. It will be used to document cigarette, tobacco, electronic nicotine delivery system (ie, e-cigarette), cannabis, any other substance uses, and TUD medications. Participants who are in CM condition will be assessed at screening for past 30-day use, at weekly sessions for past 7-day or days since the last TLFB assessment, and at follow-up visit for days since the last TLFB assessment; participants who are in TAU condition will be assessed at screening for past 30-day use and at follow-up visit for days between the last TLFB assessment and follow-up assessment. Bioverification using mobile CO monitor will also be used to verify abstinence at follow-up visit. If there is inconsistency between self-report and bioverified CO level, a salivary cotinine test might be adopted as needed.

##### Nonsmoking Outcomes

The 12-Item Short Form Health Index [45] is a validated measure that assesses multiple domains of health-related quality of life; it will also be assessed at baseline and posttreatment.

### Other Data Documentations

A review of the VA medical record will be completed at baseline and posttreatment by staff, assessing medical conditions and treatment. Surgical complications will also be documented as adverse events (AEs). There are no restrictions on concomitant medical and mental health treatment. Concomitant medical and mental health treatment will be documented for all participants to track participants’ engagement in other interventions, and TAU participants will have their number of sessions in TAU interventions tracked.

### Interventions

#### TUD Medication Management for Both Conditions

A study physician will meet all participants receiving CM and TAU at baseline (week 0) to prescribe a 5-week supply of nicotine replacement therapy (NRT; patch, gum, and lozenge), bupropion, and varenicline, if clinically indicated and desired, following a standard prescribing algorithm [21]. Medication for individuals with TUD is recommended (unless medically contraindicated) but will not be required for study participation.

#### CM Condition: Overview and Timeline

The CM counseling intervention is based on the VA Center of Excellence in Substance Addiction Treatment and Education CM residential treatment protocol for smoking cessation [26,27], adapted in three ways: (1) CM incentives are introduced after 1 week of practice, and the intervention duration is extended (ie, from 4 to 5 wk), (2) mobile CO bioverification is used to address the barrier of requiring frequent in-person visits, and (3) a fixed rather than variable schedule incentive structure is used to enable remote bioverification. The intervention timeline includes week 1 preparation for quit date, week 2 quit date and CM initiation, weeks 3 to 4 CM abstinence reinforcement, and week 5 CM relapse prevention. Table 2 depicts the components in this mCM protocol.

Bioverification will occur through the use of a mobile CO monitor and app (iCO Smokerlyzer, CoVita Bedfont [46]). Participants will upload a daily video of themselves performing the CO test with the given CO monitor to a secure VA platform, for clinical purposes only. iCO devices are intended for personal use only and will be provided to each participant.

During week 1, the training and preparation phase, participants will receive guides and training sessions. They will either use a study iPad provided by research staff or proceed with their own electronic device to record themselves performing the CO test, exhaling into the device and showing the CO reading on camera. The CO video upload will occur once per day, with weekends and holidays being optional. The daily CO video will be uploaded to My VA Images, a secure VA platform, where veterans can share videos and photos securely with staff. Participants will receive US $5 per day for the daily video submission in week 1 irrespective of the CO reading (ie, not contingent on abstinence).

During weeks 2 to 5, the intervention phase, participants will upload videos verifying smoking abstinence at a minimum of once per day and 5 times per week, with weekends and holidays being optional. Abstinence is defined by CO levels ≤6 parts per million (ppm) [47]. An escalating monetary reinforcement schedule for the proof-of-abstinence video uploads will follow a previously published CM protocol [26,27], ranging from US $5 to approximately US $45, adapted to include a nominal incentive for video uploads. The incentive will reset to US $5 following a positive CO reading or missed video upload without a documented excused absence. The salivary cotinine test will be used as needed to resolve inconsistencies between self-reported abstinence and the CO results. Besides monetary reinforcement, clinician feedback will be provided after each CO reading upload, following previously established VA protocols for CM [25]. CO readings will be reinforced with weekly feedback sessions from study clinicians, which will be incorporated into behavioral coaching/counseling sessions. Feedback sessions will be scripted to encourage continued abstinence through nonmonetary positive reinforcement, including assessments of urges/cravings and use behaviors, and expressions of empathy and confidence [25]. Participants can earn approximately US $595 maximum for uploading daily videos throughout 5 weeks and maintaining abstinence from weeks 2 to 5. Compensation rates are approximate and will be raised as needed to account for inflation, aligned with practices recommended by VA CM subject-matter experts at the Center of Excellence in Substance Addiction Treatment and Education.

For participants who are also using noncigarette tobacco or e-cigarettes, abstinence cannot be bioverified. In these instances, participants will be educated about the risks of these products, and their use will be tracked in the TLFB [43,44] but will not determine CM incentives. Technology reluctance is relatively common among older adult outpatients and can be a barrier to telehealth interventions [48]. To promote adherence, we will order a technology-support consult for individuals with identified technology reluctance or technical challenges and will meet with participants until they gain proficiency with video uploads. For those who are unable to upload videos after these efforts, staff will meet via secure video conference to capture and record CO readings on behalf of the participants.

#### CM Condition: Behavioral Counseling

Participants will receive a 5-session smoking cessation behavioral counseling program lasting approximately 15 to 20 minutes weekly. Sessions may be conducted through secure video conference or by telephone, depending on participant preference. Sessions will incorporate cognitive behavioral therapy along with motivational interviews to resolve reluctance to quit. Counseling will include health education and goal setting for smoking cessation, coping with withdrawal symptoms, relapse prevention skills, and as-needed motivational interviewing techniques to target reluctance about cessation. The recommended quit date is in week 2, after preparatory steps to quit are introduced in week 1. The behavioral protocol will be a 5-session adaptation of the VA Integrated Care for Smoking Cessation protocol [49].

#### TAU Condition

Control participants will receive usual care at the SFVAHCS: (1) referral to the Tobacco Cessation Clinic and (2) referral to the VA’s free Telephone Quitline, “TeleQuit” (known as “Quit VET”). The Tobacco Cessation Clinic is a consult service that proactively calls patients 3 times to offer pharmacological and behavioral smoking cessation treatment. Veterans will also be provided with information on accessing Quit VET, a national toll-free number available to veterans that allows them to speak with a smoking cessation counselor for up to 5 sessions [50]. At week 6, participants receiving TAU will document their participation in these interventions via self-report, and a medical chart review will be conducted to collect information regarding their engagement.

### Outcomes Evaluation

#### Primary Outcomes: Feasibility

We will evaluate feasibility by the following: (1) Recruitment rate: whether at least 25 of 36 participants (≥70% of the target sample) are enrolled within the study period. (2) Level of engagement: engagement in CM will be evaluated by the documented proportion of CO videos uploaded in weeks 2 to 5 among those randomized to CM. (3) Retention: completion rate of study measures at posttreatment among eligible participants will be assessed.

#### Primary Outcomes: Smoking Cessation

Frequency of quit attempts will be assessed using NTUS and TLFB, defined as a period of intentionally not smoking for ≥24 hours. Number of cigarettes per day will be assessed by TLFB; nicotine dependence severity will be assessed via FTND.

#### Exploratory Outcomes

Seven-day point prevalence of cigarette abstinence will be assessed post treatment, defined as (1) no smoking for 7 days prior (via TLFB) and (2) CO levels ≤6 ppm. For individuals with CO levels >6 ppm, who report smoking cannabis via TLFB and are not receiving NRT, salivary cotinine <10 ng/ml will be used [47,51]. Cutoffs are recommended by the SRNT (Society for Research on Nicotine and Tobacco) guidelines [47].

### Data Analytic Plan and Monitoring

The results will be reported using CONSORT (Consolidated Standards of Reporting Trial) guidelines [52], and analyses will be conducted using IBM SPSS and R software (R Core Team, R Foundation). Analyses will employ an intent-to-treat approach, which includes all randomized participants regardless of treatment adherence. As-treated analyses will also be conducted to examine change in smoking cessation outcomes as a function of treatment adherence. The procedure for as-treated analyses will include intervention adherence variables (ie, feedback/counseling session attendance and medication adherence) and their interactions with treatment conditions as predictors in the models. Continuous outcome variables will be checked for normality, homoskedasticity, linearity, and missingness. Descriptive analyses, repeated measures ANOVA (rmANOVA), and generalized linear mixed models (GLMMs) will be conducted to confirm study feasibility (aim 2). Descriptive analyses will include bivariate associations between demographic variables, time, and outcome variables (recruitment, engagement, and retention) via chi-squared tests, *t* tests, and correlations. rmANOVA will use time (2 levels: baseline and end-of-treatment) as the predictor. GLMMs will be used to analyze CM attendance and video uploads, in addition to intervention efficacy (aim 3*),* and posttreatment 7-day point prevalence of cigarette abstinence. Differences between treatment groups on quit attempts, mean cigarettes per day, and severity of nicotine dependence across time will be analyzed through GLMMs, with predictors of time, intervention condition, and the time-by-treatment interaction, implemented via the *lme4* and *WeMix* packages in R [53,54].

Sensitivity analyses will be used to test the influence of missing data and the assumptions related to missing data. If there is substantial missingness or attrition (>10%), we will address the missing items using multiple imputation via the *mice* package in R and conduct sensitivity analyses incorporating “inverse-probability of censoring” weights to account for attrition.

### Power Analysis

Because the nature of aim 2 was primarily to examine feasibility, we aimed only to observe large effects (*f*=.40) with a modest degree of power (β=.80). The results of the power analysis based on rmANOVA with α=.05 indicate that the proposed sample size (n=18 per condition) is adequate for proposed analysis (minimum n=16). Because intervention efficacy is an exploratory aim in this pilot test, we are not attempting to power the analyses for aim 3.

### Oversight, Monitoring, and Stopping Rules

Each week, research staff will check in with CM participants via Questionnaire of Smoking Urges item and TLFB. Research staff will also assess CM participants for AEs and serious adverse events (SAEs) weekly and report all AEs to the PI per local institutional review board (IRB) guidelines and in accordance with ClinicalTrials.gov standards [55]. All participants will be assessed for AEs at week 0 and week 6. For any participants who wish to withdraw during the study, reasons for withdrawal will be documented, if available. Participants who have lost contact after an expected contact or visit for at least 1 month will be withdrawn from the study. Exceptions may be made after discussions with the PI, in consultation with the Data Safety Monitoring Board (DSMB), and in consistency with the regulations of IRB.

### CM Training, Supervision, and Fidelity Monitoring

Study investigators, who have demonstrated competency in national VA CM training rollouts [34], will train therapists and staff. The CM feedback sessions will be audio-recorded for the purpose of weekly supervision and fidelity monitoring. Approximately 10% of recordings will be randomly selected to rate adherence using the modified CM Competence Scale for Reinforcing Abstinence [25], a validated 12-item scale that measures adherence to CM principles in bioverification, assessing the salience of reward, praising abstinence, assessing cravings or urges, and empathy. All data collected will be entered into and stored in an encrypted Excel file, an Access database, and VA REDCap behind the VA secure firewall. The PI and the lab manager will review and perform data fidelity checks at least once a month.

### Composition of the Data Monitoring Committee

The DSMB is a group of clinical researchers who are not part of the investigative team. The DSMB will meet semiannually to review data reports prepared by the PI regarding the progress of the study and will monitor enrollment, retention, outcomes, AEs, and other issues related to patient safety. The DSMB will make recommendations to the PI as to whether the study should continue, be modified, or be terminated.

### AE Reporting and Harms

Research staff will document all AEs and SAEs and review them with the PI as described. All AEs and SAEs will be followed until resolution or until they are deemed to be chronic by the PI or until the participant is lost to follow-up.

### Ethical Considerations

The study was conducted in accordance with the Declaration of Helsinki and was approved by the IRB of the University of California, San Francisco (UCSF; IRB number 24‐41632; date of approval: September 16, 2024) and SFVAHCS Research Administration (reference number HERE-0017). A recent annual IRB continuing review was performed and approved on August 22, 2025. Any changes in the study protocol will be submitted to the UCSF IRB for review and approval, and the approved changes will be updated accordingly on ClinicalTrials.gov and submitted to DSMB.

Veterans will be mailed or emailed IRB-approved 2-week opt-out letters, be directly referred by clinicians, or directly refer themselves. Interested veterans will initially be screened for eligibility in person or over the phone using a brief screening checklist. Prospective participants will then meet with a team member to complete written informed consent in person at the San Francisco VA Medical Center or virtually via DocuSign, Inc, and proceed with screening. Study ID numbers will be assigned to each participant to protect their data privacy.

## Results

### Status of This Study

This pilot trial has been funded since July 2024, and a 1-year no-cost extension has been granted through June 2027. The recruitment and data collection started in December 2025. The first participant consented on December 12, 2026. As of May 2026, 18 participants have been enrolled and randomized in this pilot trial so far. The enrollment of the last participant is expected to be in March 2027 to reach a target of 36 enrolled participants, and the data collection is expected to be completed by April 2027. There will be no interim data analysis, but preliminary analysis is expected to be completed by August 2027, and all data analyses are expected to be completed by the end of 2027.

### Dissemination and Data Sharing Plan

The results will be disseminated through abstracts, presentations, and manuscript submissions. Findings will be published on ClinicalTrials.gov at the end of the study. The results will be shared in collaboration with the project community advisory panel, a multidisciplinary group of VA policymakers, and clinicians with subject matter expertise in TUD, behavioral treatment of SUD, surgery, and anesthesia. Analytical codes and the established mCM manual will be shared on the Open Science Framework. Study data will not be shared with the public but will be processed upon request in accordance with the VA data privacy policy.

## Discussion

Given the critical link between cigarette smoking and surgical outcomes and the high prevalence of smoking in those seeking major elective surgeries, we aim to adapt and test a brief, rigorous, CM smoking-cessation protocol in a perioperative setting for veterans undergoing major elective surgeries. We aim to extend established CM protocols and remain consistent with gold-standard CM practices using remote bioverification, a fixed incentive structure, and consulting with subject matter experts in the development of the protocol.

Potential barriers to success may include issues such as technology or internet issues, a high frequency of bioverification, an inability to bioverify for the use of electronic cigarettes and noncigarette tobacco products, and the heterogeneity of the surgical population [48,56]. To mitigate selection bias and attrition, our strategies will include 1 week of training and practice, ordering digital-support consults as needed and video conferencing with staff to obtain CO readings if needed. Additionally, the short half-life of CO poses a challenge to identifying a feasible, outpatient bioverification schedule that confirms daily smoking abstinence. VA protocols for smoking cessation were designed for residential treatment settings, with daily CO monitoring conducted on-site. One challenge of including participants who use smokeless tobacco, e-cigarettes, and other tobacco products is that we cannot bioverify abstinence from these products using CO monitoring. We will focus on smoking cessation bioverification and collect data pertaining to the cessation of other nicotine and tobacco products through TLFB. The CO abstinence verification plan implemented in this CM protocol aims to strike a balance between feasibility and scalability in outpatient settings and specificity in identifying bioverified abstinence from combustible cigarettes only. Our group operationalized the standard definition of smoking abstinence as self-report verified by CO≤6 ppm, as is recommended by SRNT consensus guidelines [47]. The 5-days-per-week, once-per-day CO video upload schedule includes same-day, rapid clinician feedback, which is a critical component of CM [25]. The protocol allows for optional weekend and holiday videos as well, but those videos cannot be reviewed and addressed on the same day due to outpatient staffing. The short half-life of CO and the challenge of demonstrating durable abstinence is an inherent limitation of any smoking cessation CM protocol in which nicotine replacement products are offered to be prescribed (preventing the use of cotinine testing among those prescribed NRT). Once-daily CO monitoring has been considered adequate for most smoking cessation protocols, including those in VA residential treatment programs.

Finally, surgical procedures may vary in terms of requiring vs recommending smoking abstinence preoperatively. However, for this pilot study, the type of surgery will be inclusive to facilitate recruitment and generalizability, and we will use systematic data queries to minimize selection bias in recruitment.

This project will elucidate the feasibility and efficacy of an mCM-for-smoking-cessation outpatient protocol in veterans undergoing major elective surgery. Given the negative impact of smoking on surgical outcomes and the unique window of opportunity for intensive, focused intervention afforded perioperatively, we expect this program to have high potential utility to improve surgical outcomes and reduce the burden of smoking-related disease.

## Supplementary material

10.2196/97779 Checklist 1 SPIRIT 2025 checklist.

10.2196/97779 Peer Review Report 1 Peer-review report from the California Tobacco-Related Disease Research Program (TRDRP, USA).

## Abbreviations

AE: adverse event
CM: contingency management
CO: carbon monoxide
CONSORT: Consolidated Standards of Reporting Trials
DSMB: Data Safety Monitoring Board
FTND: Fagerström Test for Nicotine Dependence
GLMM: generalized linear mixed model
IRB: institutional review board
mCM: mobile contingency management
NRT: nicotine replacement therapy
PGSI: Problem Gambling Severity Index
PI: principal investigator
ppm: parts per million
RCT: randomized controlled trial
rmANOVA: repeated measures ANOVA
RR: relative risk
SAEs: serious adverse events
SFVAHCS: San Francisco Veterans Affairs Health Care System
SPIRIT: Standard Protocol Items: Recommendations for Interventional Trials
SRNT: Society for Research on Nicotine and Tobacco
SUD: substance use disorder
TAU: treatment as usual
TLFB: Timeline Followback
TUD: tobacco use disorder
UCSF: University of California, San Francisco
VA: Veterans Affairs
