# Supplementary material for: Contingency Management for Veteran Smokers Undergoing Elective Major Surgery: Protocol and Rationale for a Pilot Randomized Controlled Trial
Source: JMIR Res Protoc. 2026 Sep 10;15:e97779. doi: 10.2196/97779 (PMC13561196; doi:10.2196/97779)
Supplement: Supplementary file 1 [file resprot-v15-e97779-s002.pdf]

Summary Statement - T34IP8143

Applicant Full Name: Ellen Herbst

Institution: University of California, San Francisco

Application ID: T34IP8143

Peer Review Panel: TRDRP 2024 SocBeh - Pilot Award

Application Title: Contingency Management for Veteran Smokers Undergoing Major Elective Surgery

Average Score:

1.71

Component Score Q1 (Tier 1): 1.69
Component Score Q2 (Tier 1): 2.00
Component Score Q3 (Tier 1): 1.00

Critiques:

Primary Reviewer

Critique Text: This pilot study will develop a new contingency management (CM) smoking cessation intervention to be testing in veterans undergoing major elective surgery. The use of remote biochemical verification of abstinence in this population is innovative and pending surgery represents an important teachable moment for smoking cessation. The study is very well-designed, methodologically rigorous, and has aims appropriate for a pilot study (e.g., feasibility, acceptability, engagement). The use of treatment-as-usual control group is appropriate. The investigative team is outstanding and has complementary expertise and strong letters of support from a diverse group of key stakeholders for the CAB and DSMB. The community engagement plan is not perfunctory but seems to actually have great potential to improve the study design and implementation via external feedback, as well as set the team up to broadly disseminate their research results and potential impact. These collaborators and consultants will both improve the quality of the pilot study and increase the chances of follow-up studies having wider impact. The VA is an ideal setting to test CM as there is already a robust CM infrastructure (E.g., for stimulant use disorders partnering with VA Canteen for prize-vouchers) as well as remote-monitoring/telehealth infrastructure. The application very nicely balances concerns related to internal vs external validity and appropriately justifies their design decisions in this context. Minor weaknesses include the use of cash-incentives instead of vouchers (generalizability to real-world clinical settings), the short-term follow up after surgery may lead to high relapse rates, and engagement estimates seem low. These weaknesses are relatively minor and do not significantly dampen

enthusiasm for this well designed and written application.

Critique Questions:

Q1: Please score and comment on: • Responsiveness to Intent of the Award Type: Does the applicant provide information on how the study will gather preliminary data that addresses one or more TRDRP research priorities and demonstrates proof-of-principle to support the feasibility of a new paradigm or research hypothesis? Does the study represent a new research trajectory that is not currently funded from other sources? Does the applicant describe how the pilot study will lead to an expanded research effort in the future, including specific funding sources and award types? • Tobacco-relatedness: To what extent does the application focus on tobacco prevention, treatment, regulation, or tobacco-related disease? See Appendix A of the Call for Applications for a detailed description of TRDRP Research Priorities. • Innovation: Does the research adapt existing methods or technologies to new uses or with understudied populations, propose new paradigms, challenge existing paradigms or is it otherwise highly creative in one or more of the following ways: the concept or question, research methods or technologies? Does the proposed research represent more than an incremental advance upon published data? For example, does the project challenge existing interventions, clinical practice or policy; or address an innovative hypothesis or critical barrier to progress in the field?
Weight: 30%
Answer: This is appropriate scope for a pilot award mechanism. The teams previous research suggest it is likely feasible - but that is the point of the study – to find out if in fact it is. It is certainly tobacco-related and addresses several priority populations directly (Veterans) or in a sub-population of the study sample (e.g., those seeking gender-affirming care). The study is innovative in its use of remote CO-monitoring for the specific population of pre/post surgery. The pilot will set up the team to follow up with a larger scale RCT. If successful, this could have broader implications for expanding tobacco treatment within the VA generally (e.g., CM not connected to surgery) as well as to tobacco treatment in non-VA settings.

Q2: Please score and comment on: • Significance: Does this study address an important problem? If the aims of the application are achieved, how will they advance scientific knowledge or clinical practice? What will be the effect of these studies on the concepts, methods, technologies, treatments, services or preventative interventions that drive the field of tobacco-related research or any of the stated research priorities? • Approach: Are the conceptual framework, design (including composition of study population and strength of recruitment plan), methods and analyses adequately developed, well-integrated and appropriate to the aims of the project? Has the applicant adequately considered whether the sex of an animal model or human subject should be considered a biological variable in designing their experiments? Does the applicant acknowledge potential problem areas and consider alternative strategies? • Near-term leveraging potential: At the completion of the study, is it likely that the results will be sufficiently compelling to secure follow-on

funding? How likely is it that the applicant will leverage funding from other sources to further develop this area of research within two to three years after initial funding?
Weight: 50%
Answer: Smoking treatment surrounding elective surgeries remains an important clinical need. It is important to support patients to quit prior to surgery to improve outcomes and reduce complications. Remote CM represents an important potential way to increase abstinence in this context. The study design and approach is very strong and methodologically rigorous. The potential difficulties, limitations, and alternatives are well thought out and appropriately addressed. I appreciate the candid acknowledgement of key limitations and the rationale for the design decision. The investigators place a reasonable balance on internal vs external validity in this pilot study. Implementation within VA healthcare delivery setting allows many real-world issues to be addressed in the study as they would be in clinical practice in VA, although this would not necessarily port to other healthcare settings (e.g., private insurers aren't usually mailing free iPads for telehealth visits like VA does). The outcomes and endpoints are appropriate for a pilot study that aims to develop the intervention and assess feasibility and engagement. One unique feature of CM within VA is the ability to leverage the VA Canteen as a partner for voucher-based reinforcements. It seems the current study will use cash financial incentives instead, which does raise the question of scalability and generalizability beyond the research setting into real world clinical settings (even in VA). With the intervention ending so soon after surgery, there is concern of high rates of smoking relapse after CM treatment ends. This is probably reasonable for the pilot study, but the investigators may consider extending CM for longer duration after surgery for the planned follow up larger trial? If this pilot is successful, it would position the team well to apply for a larger NIH-R01, VA-MERIT, or TRDP Research Award.

Q3: Please score and comment on: • Investigators: Are the investigators appropriately trained and well-suited to carry out this work? Is the work proposed appropriate to the experience level of the PI and other researchers? Does the investigative team bring complementary and integrated expertise to the project (if applicable)? • Environment: Does the scientific environment in which the work will be done contribute to the probability of success? Do the proposed studies benefit from unique features of the scientific environment, or subject populations or employ useful collaborative arrangements? Is there evidence of institutional support? • Community Engagement Plan: Does the applicant propose a sound approach to engaging communities affected by tobacco use in either a collaborative partnership or by proactively informing about the nature and significance of the research outcomes? To what extent does the dissemination of relevant results of funded research go beyond the research community and include channels and tools targeting clinicians, public health practitioners, advocates, policymakers and the general public?
Weight: 20%
Answer: The team of investigators has all the necessary clinical and research expertise to execute this project. The investigators include addiction psychiatry and psychology, surgery, CM, and biostats. They have a history of successful collaboration, and this is a natural extension of their expertise and related work. The institutional environment at VA/UCSF is excellent and the national partners within VA including CM content experts and tobacco treatment policy offices offer excellent avenues to inform the study design/implementation as well as for the study to be disseminated widely within VA circles after completion. The CAB has excellent representation of key stakeholders including peer support,

intervention content experts, and national program office. Letters of support indicate a very thorough and well supported study from internal and external collaborators.

Unscored Mandatory Questions:

Q1: Please comment on (Primary/secondary reviewers only; other roles type “N/A”): • Budget and out-of-state expenses: Is the budget request for the project appropriate? Is there scientific or budgetary overlap with other active awards? If out-of-state contracts or collaborations are included in the budget, how essential are they to successful completion of the project?
Answer: Budget is appropriate for the scope of the work.

Q2: Please comment on (Primary/secondary reviewers only; other roles type “N/A”): • Protection of human subjects from research risk: If human subjects are involved, will protections from research risk relating to their participation in the proposed research be assessed. • Inclusion of women, minorities and children in research: If human subjects are involved, are there adequate plans to include subjects of both genders, all racial and ethnic groups (and subgroups) and children, as appropriate for the scientific goals of the research? Are plans for the recruitment and retention of human subjects adequate? • Care and use of vertebrate animals in research: if vertebrate animals are involved in the project, plans for their care and use will be assessed. • Biohazards
Answer: The human subjects protection plan is comprehensive and appropriate. This is a small pilot so the sample size is small, but inclusion of women and minorities is appropriate relative to the Veteran population.

Secondary Reviewer

Critique Text: The PI and team propose a pilot to examine the use of a 5-week contingency management (CM + Medication) or treatment as usual (TAU + Medication) RCT among 36 Veteran smokers having major elective surgery. Aim 1 is the development of the RCT, but seems a bit unclear given the outline of the RCT components in the approach. Aim 2 assess the feasibility of the CM + Medication (outcome = 70% or more of sample), engagement in the intervention (outcome = 50% of CM + Medication completing 25% or more of CO videos/uploads/sessions), and retention (outcome = more than 60% of all participants at week 5). Aim 3 compares cessation outcomes (outcome = cigarettes per day, quit attempts, nicotine dependence scores) vs TAU at week 5. Overall, the proposal is well written, organized, and use of the mobile/remote technologies is a strength. Potential but addressable limitations include the co-use of non-combustible products among study participants, lack of clarity in any potential differences NRT vs medication (e.g., varenicline) impact on study outcomes, and the use cotinine kits if the participant is prescribed NRT.

Critique Questions:

Q1: Please score and comment on: • Responsiveness to Intent of the Award Type: Does the applicant provide information on how the study will gather preliminary data that addresses one or more TRDRP research priorities and demonstrates proof-of-principle to support the feasibility of a new paradigm or research hypothesis? Does the study represent a new research trajectory that is not currently funded from other sources? Does the applicant describe how the pilot study will lead to an expanded research effort in the future, including specific funding sources and award types? • Tobacco-relatedness: To what extent does the application focus on tobacco prevention, treatment, regulation, or tobacco-related disease? See Appendix A of the Call for Applications for a detailed description of TRDRP Research Priorities. • Innovation: Does the research adapt existing methods or technologies to new uses or with understudied populations, propose new paradigms, challenge existing paradigms or is it otherwise highly creative in one or more of the following ways: the concept or question, research methods or technologies? Does the proposed research represent more than an incremental advance upon published data? For example, does the project challenge existing interventions, clinical practice or policy; or address an innovative hypothesis or critical barrier to progress in the field?

Weight: 30%

Answer: Responsiveness to intent of the award type: This study aims to pilot a contingency management (CM) intervention of Veterans undergoing major elective surgery. Patients will be randomized to either 5-week CM with mobile CO monitoring (plus cessation medication) or treatment as usual (treatment referral, quit line, and cessation medication). Overall, responsiveness to this proposal is strong. Tobacco-relatedness: Tobacco-relatedness is strong as the proposal aims to develop a pilot RCT to examine CM with biochemical verification of cessation vs treatment as usual. Innovation: Use of mobile technologies (daily mobile CO monitoring) is innovative and strengthens the proposal.

Q2: Please score and comment on: • Significance: Does this study address an important problem? If the aims of the application are achieved, how will they advance scientific knowledge or clinical practice? What will be the effect of these studies on the concepts, methods, technologies, treatments, services or preventative interventions that drive the field of tobacco-related research or any of the stated research priorities? • Approach: Are the conceptual framework, design (including composition of study population and strength of recruitment plan), methods and analyses adequately developed, well-integrated and appropriate to the aims of the project? Has the applicant adequately considered whether the sex of an animal model or human subject should be considered a biological variable in designing their experiments? Does the applicant acknowledge potential problem areas and consider alternative strategies? • Near-term leveraging potential: At the completion of the study, is it likely that the results will be sufficiently compelling to secure follow-on funding? How likely is it that the applicant will leverage funding from other sources to further develop this area of research within two to three years after initial funding?

Weight: 50%

Answer: Significance and approach: Overall, the development of a mobile CM intervention that incorporates remote biochemical verification and check-in/feedback coupled with medication/NRT has strong potential to be an impactful study to improve smoking cessation among Veterans, and in this

case, Veterans undergoing elective surgery. In general, the approach is clear and well written. However, a few components are unclear and raise potential weaknesses in the proposal. First, the use of non-combustible products is not listed as an exclusion criterion. The use of TLFB to report use is acceptable, but unclear why, given the pilot nature of this study, individuals who use these products are included in the sample. The justification is not strong either way. The use of NRT is also something that is unclear in terms of the impact this may have on the cotinine kits for individuals who use cigarettes and cannabis, which I think is the only subpopulation of this trial who would receive the kits. If the participant is prescribed NRT as part of the RCT, how will this be handled with the cotinine test kits? Unclear how the variation of NRT and/or cessation medication will be handled in analyses (e.g., NRT, varenicline). Near-term leveraging potential: The infrastructure, team, and remote approach to this pilot study are strengths for the near-term leveraging potential.

Q3: Please score and comment on: • Investigators: Are the investigators appropriately trained and well-suited to carry out this work? Is the work proposed appropriate to the experience level of the PI and other researchers? Does the investigative team bring complementary and integrated expertise to the project (if applicable)? • Environment: Does the scientific environment in which the work will be done contribute to the probability of success? Do the proposed studies benefit from unique features of the scientific environment, or subject populations or employ useful collaborative arrangements? Is there evidence of institutional support? • Community Engagement Plan: Does the applicant propose a sound approach to engaging communities affected by tobacco use in either a collaborative partnership or by proactively informing about the nature and significance of the research outcomes? To what extent does the dissemination of relevant results of funded research go beyond the research community and include channels and tools targeting clinicians, public health practitioners, advocates, policymakers and the general public?
Weight: 20%
Answer: Investigator: PI is a clinical scientist, psychiatrist, and Chief of Addiction Recovery Treatment Services. The PI has 17+ years of expertise in this population and in addiction treatment/research/services. Environment: The environment, research program, and healthcare system are strengths. The PI/team has expertise and letters of support are strong. Community Engagement Plan: The use of a community advisory panel is a strength and would all be stakeholders interested and invested in the development of a fully powered RCT and dissemination of study findings.

Unscored Mandatory Questions:

Q1: Please comment on (Primary/secondary reviewers only; other roles type “N/A”): • Budget and out-of-state expenses: Is the budget request for the project appropriate? Is there scientific or budgetary overlap with other active awards? If out-of-state contracts or collaborations are included in the budget, how essential are they to successful completion of the project?
Answer: Budget request is appropriate. No overlap. No out of state contracts.

Q2: Please comment on (Primary/secondary reviewers only; other roles type “N/A”): • Protection of human subjects from research risk: If human subjects are involved, will protections from research risk relating to their participation in the proposed research be assessed. • Inclusion of women, minorities and children in research: If human subjects are involved, are there adequate plans to include subjects of both genders, all racial and ethnic groups (and subgroups) and children, as appropriate for the scientific goals of the research? Are plans for the recruitment and retention of human subjects adequate? • Care and use of vertebrate animals in research: if vertebrate animals are involved in the project, plans for their care and use will be assessed. • Biohazards
Answer: Protection of human subjects: Protection of human subject is appropriate. Use of HIPAA compliant portal to upload CM videos is appropriate. No personal identifiers will be used in CO readings. Care and use of vertebrate animals: N/A. Biohazards: N/A

Tertiary Reviewer

Critique Text: N/A

Critique Questions:

Q1: Please score and comment on: • Responsiveness to Intent of the Award Type: Does the applicant provide information on how the study will gather preliminary data that addresses one or more TRDRP research priorities and demonstrates proof-of-principle to support the feasibility of a new paradigm or research hypothesis? Does the study represent a new research trajectory that is not currently funded from other sources? Does the applicant describe how the pilot study will lead to an expanded research effort in the future, including specific funding sources and award types? • Tobacco-relatedness: To what extent does the application focus on tobacco prevention, treatment, regulation, or tobacco-related disease? See Appendix A of the Call for Applications for a detailed description of TRDRP Research Priorities. • Innovation: Does the research adapt existing methods or technologies to new uses or with understudied populations, propose new paradigms, challenge existing paradigms or is it otherwise highly creative in one or more of the following ways: the concept or question, research methods or technologies? Does the proposed research represent more than an incremental advance upon published data? For example, does the project challenge existing interventions, clinical practice or policy; or address an innovative hypothesis or critical barrier to progress in the field?
Weight: 30%
Answer: • Well-designed pilot study is highly responsive to the TRDRP priority of development of treatment therapies and tobacco use cessation in tobacco priority population of military veterans • Innovative – would be the first to design and test a mobile contingency management (CM) smoking cessation program for veteran smokers undergoing major elective surgery • Would also be the first to pilot remote bioverification using mobile technology for surgical patients

Q2: Please score and comment on: • Significance: Does this study address an important problem? If the aims of the application are achieved, how will they advance scientific knowledge or clinical practice? What will be the effect of these studies on the concepts, methods, technologies, treatments, services or preventative interventions that drive the field of tobacco-related research or any of the stated research priorities? • Approach: Are the conceptual framework, design (including composition of study population and strength of recruitment plan), methods and analyses adequately developed, well-integrated and appropriate to the aims of the project? Has the applicant adequately considered whether the sex of an animal model or human subject should be considered a biological variable in designing their experiments? Does the applicant acknowledge potential problem areas and consider alternative strategies? • Near-term leveraging potential: At the completion of the study, is it likely that the results will be sufficiently compelling to secure follow-on funding? How likely is it that the applicant will leverage funding from other sources to further develop this area of research within two to three years after initial funding?
Weight: 50%
Answer: • Highly significant – high prevalence of perioperative smoking, (22.3-43.0%), veterans in particular • Smoking at the time of surgery is modifiable and is associated with postoperative morbidity and mortality. • Smoking cessation treatment is not regularly integrated into surgical care. • The approach is well developed and significant in that it uses contingency management (CM) – a protocol that provides financial rewards for quitting with required bioverification to confirm abstinence. This study would be the first to integrate CM in this setting (pre and post major surgery) • The applicant has identified potential problem areas including recruitment and retention and have planned for a tailored incentive structure and individual clinician feedback during bioverification • The investigators expect a low proportion of women in the study which will limit power to analyze group differences by sex. They plan to use block randomization by biological sex and conduct exploratory analyses • Findings from the RCT should be compelling in the near term and the study team plans to apply for future funding in the last year of this study to further test efficacy of the intervention among veterans undergoing major surgery

Q3: Please score and comment on: • Investigators: Are the investigators appropriately trained and well-suited to carry out this work? Is the work proposed appropriate to the experience level of the PI and other researchers? Does the investigative team bring complementary and integrated expertise to the project (if applicable)? • Environment: Does the scientific environment in which the work will be done contribute to the probability of success? Do the proposed studies benefit from unique features of the scientific environment, or subject populations or employ useful collaborative arrangements? Is there evidence of institutional support? • Community Engagement Plan: Does the applicant propose a sound approach to engaging communities affected by tobacco use in either a collaborative partnership or by proactively informing about the nature and significance of the research outcomes? To what extent does the dissemination of relevant results of funded research go beyond the research community and include channels and tools targeting clinicians, public health practitioners, advocates, policymakers and the general public?
Weight: 20%
Answer: • Strong expert investigatory team with complementary expertise with tobacco and other substance use disorder, Veteran populations, and cessation programs with this population • Clinical

Advisory Panel of collaborators providing in-kind specialized assistance as VA experts • Community Advisory Panel of content experts to review protocol and advise on implementation and dissemination • Will conduct targeted recruitment in surgical clinics serving transgender and nonbinary surgical patients as well members of other unrepresentative minority group patients undergoing surgery

Unscored Mandatory Questions:

Q1: Please comment on (Primary/secondary reviewers only; other roles type "N/A"): • Budget and out-of-state expenses: Is the budget request for the project appropriate? Is there scientific or budgetary overlap with other active awards? If out-of-state contracts or collaborations are included in the budget, how essential are they to successful completion of the project?
Answer: N/A

Q2: Please comment on (Primary/secondary reviewers only; other roles type "N/A"): • Protection of human subjects from research risk: If human subjects are involved, will protections from research risk relating to their participation in the proposed research be assessed. • Inclusion of women, minorities and children in research: If human subjects are involved, are there adequate plans to include subjects of both genders, all racial and ethnic groups (and subgroups) and children, as appropriate for the scientific goals of the research? Are plans for the recruitment and retention of human subjects adequate? • Care and use of vertebrate animals in research: if vertebrate animals are involved in the project, plans for their care and use will be assessed. • Biohazards
Answer: N/A
